# Placental gross shape differences in a high autism risk cohort and the general population

**DOI:** 10.1371/journal.pone.0191276

**Published:** 2018-08-22

**Authors:** Bo Y. Park, Dawn P. Misra, John Moye, Richard K. Miller, Lisa Croen, M. Dani Fallin, Cheryl Walker, Craig J. Newschaffer, Caroline M. Salafia, on behalf of the National Children’s Study Consortium

**Affiliations:** 1 Department of Mental Health, Johns Hopkins Bloomberg School of Public Health, Baltimore, Maryland, United Sates of America; 2 Wendy Klag Center for Autism and Developmental Disabilities, Department of Mental Health, Johns Hopkins Bloomberg School of Public Health, Baltimore, Maryland, United Sates of America; 3 Department of Family Medicine & Public Health Sciences, School of Medicine, Wayne State University, Detroit, Michigan, United Sates of America; 4 *Eunice Kennedy Shriver* National Institute of Child Health and Human Development, National Institutes of Health, Bethesda, Maryland, United Sates of America; 5 Department of Obstetrics/Gynecology, Pathology & Clinical Laboratory Medicine, Environmental Medicine, School of Medicine & Dentistry, University of Rochester, Rochester, New York, United Sates of America; 6 Kaiser Permanente Division of Research, Broadway, Oakland, CA; 7 The MIND (Medical Investigations of Neurodevelopmental Disorders) Institute, University of California Davis, Davis, California, United Sates of America; 8 A.J. Drexel Autism institute, Philadelphia, Pennsylvania, United Sates of America; 9 Placental Modulation Laboratory, Institute for Basic Research, Staten Island, New York, United Sates of America; 10 Placental Analytics, LLC, Larchmont, New York, United Sates of America; BC Children's Hospital, CANADA

## Abstract

A growing body of evidence suggests that prenatal environment is important in Autism Spectrum Disorder (ASD) etiology. In this study, we compare placental shape features in younger siblings of children with ASD, who themselves are at high ASD risk, to a sample of low risk peers. Digital photographs of the fetal placenta surface and of the sliced placental disk from 129 high ASD risk newborns and from 267 newborns in the National Children’s Study Vanguard pilot were analysed to extract comparable measures of placental chorionic surface shape, umbilical cord displacement and disk thickness. Placental thickness measures were moderately higher in siblings of ASD cases. The placentas of ASD-case siblings were also rounder and more regular in perimeter than general population placentas. After stratification by sex, these across-group differences persisted for both sexes but were more pronounced in females. No significant differences were observed in cord insertion measures. Variations in placental shape features are generally considered to reflect flexibility in placental growth in response to changes in intrauterine environment as the placenta establishes and matures. Reduced placental shape variability observed in high ASD risk siblings compared to low-risk controls may indicate restricted ability to compensate for intrauterine changes.

## Introduction

Autism Spectrum Disorders (ASD) are neurodevelopmental conditions that manifest as impaired social communication and the presence of restricted/repetitive behaviors and interests [1]. In United States, 1 in 68 eight year old children have ASD [2]. ASD symptoms emerge in early childhood and the associated disabilities are typically lifelong [3]. Converging evidence suggests that the changes in the brain associated with ASD are initiated during prenatal brain development [4–7] but the complete nature of these changes remains unknown.

The placenta is vital to fetal growth and development and plays a key role in maintaining the homeostasis of the intrauterine environment. Fetal and/or placental cell inflammatory response [8], gene expression [9], gene-environment interaction [10], and placental morphology [11–13] have been used as measures of abnormal placental function [14] and have been shown to be associated with reduced fetal growth and adverse neonatal outcomes, including death and neurological morbidities [15]. The human placental formation begins soon after implantation and continually modifies to accommodate the increasing demands of the fetus and can be influenced by factors such as nutrients and smoking [16]. The *ex utero* placenta is a universally accessible organ that is suitable for research into possible indicators of prenatal environment, however, the placenta is generally discarded as medical waste.

A typical placenta is described as round or oval but can have various shapes [17]. Variant shapes, cord insertion sites and placental disk thickness (reflecting later villous branching arborization) have been associated with reduced placental efficiency [12]. Irregular placental chorionic surface shapes have been linked to low birthweight [18], however, it is also rare to have perfectly round placentas with central umbilical cord insertion [19] and placental shape variability may be the norm [12]. Examination of placental morphology has also yielded insights into mechanisms of pre-eclampsia, prematurity and fetal growth restriction [13, 20], which are conditions also associated with increased ASD risk [21]. Increased ASD risk have been associated with indicators of decreased placental function, such as abnormal placental pathology (trophoblast inclusion) [22] and obstetric complications (preeclampsia/eclampsia) [23].

Here we compare placental shape features in a cohort of children at higher risk for ASD, by virtue of the fact that they have older ASD-affected siblings, to a population of typical-risk peers.

## Materials and methods

### Sample collection

The National Children's Study (NCS) Vanguard recruited pregnant women during 2009–2010 in 7 locations in the United States (Orange County, California; Queens County, New York; Duplin County, North Carolina; Montgomery County, Pennsylvania; Salt Lake and Cache County, Utah; Waukesha County, Wisconson; multiple counties in South Dakota/Minnesota) using a multistage area probability sampling design to identify study locations (generally, counties) for house-hold based recruitment [24]. As such, the children in this sample would be expected to experience the typical ASD risk of the general U.S. population. The latest CDC prevalence studies suggest that 1.5% of US children have an ASD [2].

Formative research projects were conducted based on the NCS Vanguard study, one of which was focused on collection and analysis of placental samples. Of 831 enrolled and eligible pregnant women, 267 mothers consented and provided placental samples. However, in the pilot phase of placental collection, there was no attempt to collect demographic and perinatal data, which left a subgroup of 180 mothers with both placenta and linked demographic and perinatal data. However, in not all instances were all perinatal data available. Gestational age information, which had to be abstracted from medical records, was available for 31 participants.

The Early Autism Risk Longitudinal Investigation (EARLI) network of research sites (Southeast Pennsylvania: Drexel University, The Children’s Hospital of Philadelphia and the University of Pennsylvania; Northeast Maryland: Johns Hopkins University and Kennedy Krieger Institute; Northern California: University of California Davis) enrolled a group of mothers of children with ASD diagnoses at the start of a subsequent pregnancy and is documenting the development of the newborn child (sibling of the ASD case) through three years of age [25]. EARLI singleton birth placentas from 133 mothers were available. Four subjects were excluded because placenta morphology measures could not be obtained and129 placentas were included in the analysis. The study was approved by multiple Institutional Review Boards (IRB) listed below:

Mt. Sinai Institutional Review Boards, University of Rochester IRB, UC Davis IRB, Medical College of Wisconsin IRB, University of North Carolina IRB, University of Utah IRB, UC Irvine IRB, South Dakota State University IRB, University of Pennsylvania IRB, Northwestern University IRB, Columbia University IRB, University of Illinois at Chicago IRB, Baylor University IRB, University of Arkansas IRB, University of Iowa IRB, University of Massachusetts IRB, Duke University IRB, and Drexel University IRB.

### Placenta morphology measures

Placentas were fixed with formalin in uniform sealable bags for one week. A uniform protocol of placental gross measurement developed by the pathology group collected a range of placental gross measures that reflect three facets of placental shape: the chorionic plate surface shape and irregularity; umbilical cord displacement; and disk thickness (Table 1). Digital photographs were obtained of the placental chorionic surface shape which included the site of the umbilical cord insertion, and of the sliced placental disk. The chorionic surface shape and cord insertion, or the edge of the placental disk slice, were marked as a “layer” in GIMP (GNU Image Manipulation Program). These layers were then analyzed using dedicated algorithms as described previously [12, 26, 27]. Thickness measures could not be obtained in three EARLI and 84 NCS samples. All measurements were obtained by a trained pathologist blinded to the study group.

**Table 1:** Placenta morphology measure definitions.

Morphology	Definition
Perimeter (cm)	perimeter of the placenta from the traced 2D fetal surface image
Area (cm^2^)	area of the placenta from the traced 2D fetal surface image
Radius (cm)	radius from geometric center to the perimeter of the placental disk measured per degree
Median	median radius of the placenta based on 360 radii
Minimum	minimum radius of the placenta based on 360 radii
Maximum	maximum radius of the placenta based on 360 radii
Umbilical distance from center (cm)	distance between umbilical cord insertion and geometric center of the placenta from the traced 2D fetal surface image
Eccentricity	
Umbilical cord	maximum radius/minimum radius from cord insertion point of the disk
Geometric center	maximum radius/minimum radius from geometric center of the disk
Thickness (cm)	thickness of the placenta f each vertical pixel pair of the disk slice
Maximum	maximum thickness of the disk slice
Mean	mean thickness of the disk slice
Standard deviation	standard deviation of thickness of the disk slice

Several placental morphology measures, such as placental disk eccentricity, were sekewed. Wilcoxon rank-sum test was used to compare all perinatal characteristics and placental morphology between EARLI and NCS. Spearman’s rho was used to examine the correlation between placental shapes and perinatal characteristics. Chi squared test was used to test for difference between categorical variables. An alpha-error tolerance of 5% (*P*<0.05) was used to determine statistical significance of comparisons. Wilcoxon rank-sum test was also used to determine if differences in EARLI and NCS placentas varied significantly within sex. Two way ANOVA was used to examine whether any association between placental morphology and cohort differed by the sex of the subject. Birthweight and gestational age have been associated with placental size and ASD risk. Birthweight was not available in 8 (6%) EARLI subjects and gestational age was not available in 88% of NCS subjects. Sex was missing in one EARLI subject and was excluded from the sex tratified analysis. Spearman’s correlation was used to determine correlation between birthweight, gestational age and placental morphology. All analyses were performed using STATA 12 [28].

## Results

Siblings of children diagnosed with ASD were similar in sex ratio, birthweight, and gestational age compared to NCS subjects in the subset with perinatal data (Table 2). Correlations between birthweight and placenta morphologies were in same direction across sexes except for the umbilical distance from center (Spearman’s rho -0.12 to 0.47) with strongest correlation observed with area and birthweight among females (Spearman’s rho 0.47 [0.39, 0.58]) (Table 3). Gestational age and placental shapes correlations showed more vairation in direction and magnitude across sexes (Spearman’s rho -0.16 to 0.33) with strongest correlation observed between gestational week and median radius among males (Spearman’s rho 0.33 [0.21, 0.43]) (Table 3).

**Table 2:** Perinatal characteristics by study.

	EARLI (n = 129)					NCS (n = 267)					
	N	Mean	SD	Min	Max	n	Mean	SD	Min	Max	*P*
Birthweight (g)	121	3454	532.7	2296	6039	180	3498	430.8	2190	4760	0.49
Gestational week	127	39.46	1.3	34	42	31	39.48	1.1	37	41	0.97
Male (%)	128	49%				180	50%				0.89

SD = Standard deviation; Min = minimum; Max = maximum.

**Table 3:** Spearman’s correlation (rho) between birthweight, gestational week and placental morphology by child sex.

	Birthweight (g)				Gestational week			
	Males		Females		Males		Females	
	Rho	95% CI	rho	95% CI	rho	95% CI	rho	95% CI
Perimeter (cm)	0.34	[0.20, 0.43]	0.42	[0.33, 0.53]	0.26	[0.14, 0.37]	-0.09	[-0.21, 0.04]
Area (cm^2^)	0.37	[0.25, 0.46]	0.47	[0.39, 0.58]	0.26	[0.14, 0.37]	-0.12	[-0.23, 0.01]
Radius (cm)								
Median	0.22	[0.09, 0.32	0.31	[0.21, 0.43]	0.33	[0.21, 0.43]	-0.11	[-0.23, 0.01]
Minimum	0.16	[0.04, 0.28]	0.15	[0.06, 0.3]	-0.02	[-0.14, 0.1]	0.09	[-0.03, 0.21]
Maximum	0.06	[-0.08, 0.17]	0.22	[0.12, 0.35]	0.22	[0.1, 0.34]	-0.16	[-0.28, -0.04]
Umbilical distance from center (cm)	-0.07	[-0.21, 0.03]	0.04	[-0.08, 0.17]	0.14	[0.02, 0.26]	-0.13	[-0.25, -0.003]
Eccentricity								
Umbilical cord	-0.12	[-0.24, -0.002]	-0.04	[-0.2, 0.05]	0.07	[-0.05, 0.19]	-0.16	[-0.27, -0.03]
Geometric center	-0.05	[-0.20, 0.04]	-0.03	[-0.2, 0.05]	0.07	[-0.05, 0.19]	0.02	[-0.1, 0.14]
Thickness (cm)								
Maximum	0.06	[-0.05, 0.19]	0.14	[0.05, 0.29]	0.13	[0.01, 0.25]	-0.05	[-0.17, 0.08]
Mean	0.05	[-0.06, 0.19]	0.10	[0.05, 0.29]	0.15	[0.02, 0.26]	-0.06	[-0.18, 0.06]
Standard deviation	0.06	[-0.07, 0.18]	0.06	[-0.07, 0.18]	0.06	[-0.02, 0.22]	-0.02	[-0.14, 0.11]

EARLI placentas had smaller, but non-statistically significant differences in, median radius, umbilical cord distance from center, and eccentricity from umbilical cord compared to NCS placentas (Table 4). We did not observe statistically significant differences between the average placental disk thickness across EARLI and NCS placentas (*P* = 0.71), although maximum thickness (*P* = 0.01) and standard deviation of thickness (*P* = 0.01) were significantly greater in EARLI subjects. Finally, eccentricity, the placenta measures that capture shape deviation from a circle, based on the geometric center of the placenta, were significantly lower in EARLI than in NCS placentas (*P* = 0.04). The eccentricity using umbilical cord insertion point showed the same directionality but were not statistically significant (*P* = 0.12).

**Table 4:** Comparison of placental shape measures between EARLI and NCS using the Wilcoxson rank-sum test.

	EARLI (n = 129)				NCS (n = 267)				
	Mean	SD	Min	Max	Mean	SD	Min	Max	P*
Perimeter (cm)	61.85	7.68	45.00	92.40	61.54	6.59	48.60	93.00	0.73
Area (cm^2^)	274.40	53.04	156.00	440.00	272.10	49.76	175.00	496.00	0.62
Radius (cm)									
Median	10.09	1.23	7.40	13.90	10.13	1.27	6.97	15.80	0.85
Minimum	5.25	2.08	0.42	8.95	4.98	1.90	0.34	9.61	0.13
Maximum	13.55	2.31	9.46	19.50	13.75	2.27	8.51	22.50	0.37
Umbilical distance	3.48	2.07	0.23	8.95	3.68	1.96	0.04	10.50	0.27
from center (cm)									
Eccentricity									
Umbilical cord	4.14	5.84	1.23	40.67	4.45	7.01	1.23	51.88	0.12
Geometric center	1.40	0.26	1.11	2.64	1.45	0.33	1.11	3.51	0.04
Thickness (cm)**									
Maximum	2.34	0.34	1.71	3.76	2.24	0.33	1.52	3.52	0.01
Mean	1.74	0.27	1.26	2.78	1.72	0.27	1.08	2.87	0.71
SD	0.45	0.10	0.22	0.77	0.41	0.10	0.21	0.72	0.01

*Determined using the Wilcoxon rank-sum test.

**Thickness was available in 123 EARLI and 183 NCS samples.

SD = Standard deviation; Min = minimum; Max = maximum.

Table 5 shows the same comparisons stratified by sex. The minimum placental radius was greater in EARLI placentas compared to NCS among females (*P* = 0.05) but not in males. The eccentricity was greater in NCS among both males and females (smaller in EARLI placentas), but the difference remained statistically significant only for eccentricity from geometric center in females (*P* = 0.04). Two placental thickness measures, maximum and standard deviation, were greater in EARLI placentas compared to NCS with each sex, however, only the maximum thickness showed significant difference among females (*P* = 0.02). Two way ANOVA test of EARLI-NCS differences across sexes for any measure were not statistically significant.

**Table 5:** Comparison of placental morphology measures between EARLI and NCS by sex using the Wilcoxson rank-sum test.

	Male					Female				
	EARLI (n = 64)		NCS (n = 90)			EARLI (n = 64)		NCS (n = 90)		
	Mean	SD	Mean	SD	P*	Mean	SD	Mean	SD	P*
Perimeter (cm)	61.00	6.26	61.56	6.35	0.86	62.89	8.93	60.80	6.12	0.19
Area (cm^2^)	268.3	44.4	274.4	51.5	0.92	282.0	60.6	267.8	47.1	0.23
Radius (cm)										
Median	10.08	1.30	10.24	1.40	0.52	10.13	1.19	9.94	1.12	0.30
Minimum	4.91	2.15	4.88	1.92	0.82	5.55	1.99	5.05	1.70	0.05
Maximum	13.83	2.39	13.96	2.16	0.53	13.34	2.22	13.56	2.22	0.54
Umbilical distance from center (cm)	3.82	2.24	3.90	2.02	0.73	3.19	1.86	3.57	1.83	0.16
Eccentricity										
Umbilical cord	5.31	7.91	4.73	7.38	0.65	3.07	2.26	3.71	5.46	0.12
Geometric center	1.40	0.22	1.42	0.31	0.84	1.39	0.30	1.41	0.27	0.04
Thickness (cm)**										
Maximum	2.32	0.28	2.27	0.33	0.32	2.37	0.39	2.23	0.34	0.02
Mean	1.71	0.22	1.74	0.26	0.58	1.77	0.30	1.71	0.28	0.31
SD	0.46	0.11	0.42	0.09	0.02	0.43	0.10	0.41	0.10	0.12

*Determined using the Wilcoxon rank-sum test.

**Thickness was available among 62 male and 64 female EARLI samples.

SD = Standard deviation; Min = minimum; Max = maximum.

## Discussion

Proper placental function is essential to normal fetal development and the shape and function of the placenta can be affected by variations in maternal vascular supply, location of uterine implantation, regional variation in the decidua, and structure of underlying placental vascular tree [11]. The relationship between placental morphology and fetal neurodevelopment is largely unknown.

As calculated from a large modern birth cohort with extensive photography images, the typical placental shape is circular with a center at the umbilical insertion point [12] and irregular placental shapes have been associated with lower birthweight [27]. However, perfectly round placentas with central umbilical cord insertion are uncommon [19] and some variability of placental shapes may be the norm [12]. Interestingly, our study of placental morphology suggested reduced variability in placental chorionic surface shapes in EARLI as compared to NCS placentas. We also observed that EARLI placentas had greater maximum thickness and standard deviation compared to NCS placentas. It is important to emphasize that the observed significant differences across EARLI and NCS placental morphology were modest in magnitude compared to the observed variation of each measures. Furthermore, our understanding of connections between placental morphology and nuerodevelopent is still in the formative stages and we need additional studies to determine whether these differences are epiphenomena of a poor intratuderine environment or etiologically important mechanisms in neurodevelopment.

The placenta’s morphology at the time of delivery may provide information on etiologically significant events occurring earlier in pregnancy. Comparisons of placental morphology from detailed ultrasonography data collected at 11–14 weeks gestation and placental measures obtained at term delivery show correlations from 0.14 (Pearson’s r of umbilical cord displacement at 11–14 weeks and at term) to 0.25 (Pearson’s r of maximal placental disk thickness at 11–14 weeks and the at term) [29]. Observed differences between siblings of ASD individuals to general population in placental shapes may be reflecting differences in the maternal intrauterine environments. Towards the end of the first trimester extensive villous remodeling takes place with onset of the maternal arterial circulation. Later in pregnancy, a wide variety of stressors are capable of affecting placental growth, the most common being nutrient deprivation and vascular compromise [30]. Mothers of ASD individuals may be exposed to more gestaional stressors including inflammation and oxidative stress and/or carrie genetic risk factors that interferes with proper response to changes in intrauterine environment.

This study is a comparison of placentas from mothers of children at higher risk of ASD to placentas from a general population of mothers and not a comparison of placentas from mothers of children with and without ASD. It is important to note that the we are comparing high and low risk population and that the NCS children, although they are at low risk at developing ASD, may not all be typically developing children. The increased recurrence risk of ASD in younger siblings of older affected children mentioned above has been estimated to range from 3 to 25% [31, 32]. The latest estimate from the ASD Baby Sibs Research Consortium, a multisite study with a similar case-confirmation approach to EARLI, is at the upper end of this range [32]. Recently, evidence has been presented from a small nested case-control comparison of 52 ASD cases and 161controls from the ALSPAC (Avon Longitudinal Study of Parents and Children) general population pregnancy cohort study that suggests reduced eccentricity from cord insertion and reduced villous branching growth in those children who will receive an ASD diagnosis when compared to sex and gestational age matched peers [33]. The reduced variability in both ASD individuals and siblings of ASD may indicate a shared disadvantage of lesser adaptive capacity and greater vulnerability to gestational stressors.

Several obstetric complications that may reflect or induce an altered intrauterine environment, have been associated with ASD [23, 34–37]. Two of these ASD risk factors have been associated with both decreased placental thickness in pre-eclampsia [38] and increased thickness among intrauterine growth restriction [39]. If we assume that differences we observed in placentas from the at-risk cohort reflect differences that are relevant to ASD etiology, differences we observe in this study could be the result of obstetric complications. However, data on obstetric complications and other possible confounders in the NCS population were not available at the time of the current study and were not included in the analysis. This is a major limitation of this study and these findings must be considered as preliminary and need to be replicated.

Our findings suggest that there may be some gross morphological differences between general population and high ASD risk placentas and provide an initial indication that the ASD placenta might be less able to compensate for intrauterine variability. Morphological changes could be merely a marker for vulnerability to risk factors or it could be mechanistically important in ASD etiology if reduced compensatory capacity leaves the fetus more vulnerable to other stressors. Results need to be cautiously interpreted as this is a small initial study and multiple comparisons were done in an exploratory manner which could lead to false-positive findings. Future work should explore whether these placental morphological differences are associated with the ASD phenotype. Moreover, investigation on whether obstetric complications are associated with placental morphology would help clarify the placental morphology differences that are observed in the current study.

## References

[1] American Psychiatric Association. Diagnostic and statistical manual of mental disorders. 5th ed ed. Arlington, VA: American Psychiatric Publishing,; 2013.

[2] Centers for Disease Control and Prevention. Prevalence of autism spectrum disorder among children aged 8 years—autism and developmental disabilities monitoring network, 11 sites, United States, 2010. MMWR Surveill Summ. 2014;63(2):1–21. Epub 2014/03/29. . 24670961

[3] Fein D, Barton M, Eigsti IM, Kelley E, Naigles L, Schultz RT, et al Optimal outcome in individuals with a history of autism. J Child Psychol Psychiatry. 2013;54(2):195–205. Epub 2013/01/17. 10.1111/jcpp.12037 ; PubMed Central PMCID: PMCPmc3547539. 23320807 PMC3547539

[4] Voineagu I, Wang X, Johnston P, Lowe JK, Tian Y, Horvath S, et al Transcriptomic analysis of autistic brain reveals convergent molecular pathology. Nature. 2011;474(7351):380–4. Epub 2011/05/27. 10.1038/nature10110 ; PubMed Central PMCID: PMCPMC3607626. 21614001 PMC3607626

[5] Willsey AJ, Sanders SJ, Li M, Dong S, Tebbenkamp AT, Muhle RA, et al Coexpression networks implicate human midfetal deep cortical projection neurons in the pathogenesis of autism. Cell. 2013;155(5):997–1007. Epub 2013/11/26. 10.1016/j.cell.2013.10.020 ; PubMed Central PMCID: PMCPmc3995413. 24267886 PMC3995413

[6] Amaral DG, Schumann CM, Nordahl CW. Neuroanatomy of autism. Trends in neurosciences. 2008;31(3):137–45. 10.1016/j.tins.2007.12.005 18258309

[7] Stoner R, Chow ML, Boyle MP, Sunkin SM, Mouton PR, Roy S, et al Patches of disorganization in the neocortex of children with autism. N Engl J Med. 2014;370(13):1209–19. Epub 2014/03/29. 10.1056/NEJMoa1307491 ; PubMed Central PMCID: PMCPmc4499461. 24670167 PMC4499461

[8] Galinsky R, Polglase GR, Hooper SB, Black MJ, Moss TJ. The consequences of chorioamnionitis: preterm birth and effects on development. Journal of pregnancy. 2013;2013:412831 Epub 2013/03/28. 10.1155/2013/412831 ; PubMed Central PMCID: PMCPmc3606792. 23533760 PMC3606792

[9] Kleinrouweler CE, van Uitert M, Moerland PD, Ris-Stalpers C, van der Post JA, Afink GB. Differentially expressed genes in the pre-eclamptic placenta: a systematic review and meta-analysis. PLoS One. 2013;8(7):e68991 Epub 2013/07/23. 10.1371/journal.pone.0068991 ; PubMed Central PMCID: PMCPmc3709893. 23874842 PMC3709893

[10] Waterland RA, Jirtle RL. Transposable elements: targets for early nutritional effects on epigenetic gene regulation. Molecular and cellular biology. 2003;23(15):5293–300. Epub 2003/07/16. 10.1128/MCB.23.15.5293-5300.2003 ; PubMed Central PMCID: PMCPmc165709. 12861015 PMC165709

[11] Longtine MS, Nelson DM. Placental dysfunction and fetal programming: the importance of placental size, shape, histopathology, and molecular composition. Semin Reprod Med. 2011;29(3):187–96. Epub 2011/06/29. 10.1055/s-0031-1275515 ; PubMed Central PMCID: PMCPmc3682832. 21710395 PMC3682832

[12] Salafia CM, Yampolsky M, Misra DP, Shlakhter O, Haas D, Eucker B, et al Placental surface shape, function, and effects of maternal and fetal vascular pathology. Placenta. 2010;31(11):958–62. Epub 2010/10/12. 10.1016/j.placenta.2010.09.005 ; PubMed Central PMCID: PMCPmc2964412. 20933281 PMC2964412

[13] Salafia CM, Zhang J, Charles AK, Bresnahan M, Shrout P, Sun W, et al Placental characteristics and birthweight. Paediatr Perinat Epidemiol. 2008;22(3):229–39. Epub 2008/04/23. 10.1111/j.1365-3016.2008.00935.x . 18426518

[14] Norwitz ER. Defective implantation and placentation: laying the blueprint for pregnancy complications. Reproductive biomedicine online. 2006;13(4):591–9. Epub 2006/09/30. . 17007686 10.1016/s1472-6483(10)60649-9

[15] Roescher AM, Timmer A, Erwich JJ, Bos AF. Placental pathology, perinatal death, neonatal outcome, and neurological development: a systematic review. PLoS One. 2014;9(2):e89419 Epub 2014/03/04. 10.1371/journal.pone.0089419 ; PubMed Central PMCID: PMCPmc3934891. 24586764 PMC3934891

[16] Pereira RD, De Long NE, Wang RC, Yazdi FT, Holloway AC, Raha S. Angiogenesis in the Placenta: The Role of Reactive Oxygen Species Signaling. BioMed Research International. 2015;2015:814543 10.1155/2015/814543 PubMed PMID: PMC4325211. 25705690 PMC4325211

[17] Benirschke KK P. Pathology of the human placenta. 4th ed New York, NY: Springer; 2000.

[18] Salafia CM, Misra DP, Yampolsky M, Charles AK, Miller RK. Allometric metabolic scaling and fetal and placental weight. Placenta. 2009;30(4):355–60. Epub 2009/03/07. 10.1016/j.placenta.2009.01.006 ; PubMed Central PMCID: PMCPmc3779882. 19264357 PMC3779882

[19] Baergen RN BK. Macroscopic Features of the Delivered Placenta Manual of Pathology of the Human Placenta Springer Verlag Springer Berlin Heidelberg; 2012.

[20] Roberts JM, Escudero C. The placenta in preeclampsia. Pregnancy hypertension. 2012;2(2):72–83. 10.1016/j.preghy.2012.01.001 ; PubMed Central PMCID: PMCPMC3381433. 22745921 PMC3381433

[21] Gardener H, Spiegelman D, Buka SL. Perinatal and neonatal risk factors for autism: a comprehensive meta-analysis. Pediatrics. 2011;128(2):344–55. Epub 2011/07/13. 10.1542/peds.2010-1036 ; PubMed Central PMCID: PMCPmc3387855. 21746727 PMC3387855

[22] Walker CK, Anderson KW, Milano KM, Ye S, Tancredi DJ, Pessah IN, et al Trophoblast inclusions are significantly increased in the placentas of children in families at risk for autism. Biological Psychiatry. 2013;74(3):204–11. 10.1016/j.biopsych.2013.03.006 23623455 PMC3755347

[23] Walker CK, Krakowiak P, Baker A, Hansen RL, Ozonoff S, Hertz-Picciotto I. Preeclampsia, placental insufficiency, and autism spectrum disorder or developmental delay. JAMA pediatrics. 2015;169(2):154–62. Epub 2014/12/09. 10.1001/jamapediatrics.2014.2645 ; PubMed Central PMCID: PMCPMC4416484. 25485869 PMC4416484

[24] Baker D, Park C, Sweeney C, McCormack L, Durkin M, Brenner R, et al Recruitment of women in the National Children's Study Initial Vanguard Study. Am J Epidemiol. 2014;179(11):1366–74. Epub 2014/05/06. 10.1093/aje/kwu062 ; PubMed Central PMCID: PMCPmc4036215. 24793429 PMC4036215

[25] Newschaffer CJ, Croen LA, Fallin MD, Hertz-Picciotto I, Nguyen DV, Lee NL, et al Infant siblings and the investigation of autism risk factors. Journal of neurodevelopmental disorders. 2012;4(1):7–1955-4-7. 10.1186/1866-1955-4-7 22958474 PMC3436647

[26] Yampolsky M, Salafia CM, Shlakhter O, Haas D, Eucker B, Thorp J. Centrality of the umbilical cord insertion in a human placenta influences the placental efficiency. Placenta. 2009;30(12):1058–64. Epub 2009/11/03. 10.1016/j.placenta.2009.10.001 ; PubMed Central PMCID: PMCPmc2790011. 19879649 PMC2790011

[27] Salafia CM, Maas E, Thorp JM, Eucker B, Pezzullo JC, Savitz DA. Measures of placental growth in relation to birth weight and gestational age. Am J Epidemiol. 2005;162(10):991–8. Epub 2005/09/30. 10.1093/aje/kwi305 . 16192346

[28] StataCorp. Stata Statistical Software: Release 12. College Station, TX: StataCorp LP 2011.

[29] Salafia CM, Yampolsky M, Shlakhter A, Mandel DH, Schwartz N. Variety in placental shape: when does it originate? Placenta. 2012;33(3):164–70. Epub 2012/01/06. 10.1016/j.placenta.2011.12.002 . 22217910

[30] Burton GJ, Jauniaux E, Charnock-Jones DS. The influence of the intrauterine environment on human placental development. The International journal of developmental biology. 2010;54(2–3):303–12. Epub 2009/09/17. 10.1387/ijdb.082764gb . 19757391

[31] Sandin S, Lichtenstein P, Kuja-Halkola R, Larsson H, Hultman CM, Reichenberg A. The familial risk of autism. Jama. 2014;311(17):1770–7. Epub 2014/05/06. 10.1001/jama.2014.4144 . 24794370 PMC4381277

[32] Ozonoff S, Young GS, Carter A, Messinger D, Yirmiya N, Zwaigenbaum L, et al Recurrence risk for autism spectrum disorders: a Baby Siblings Research Consortium study. Pediatrics. 2011;128(3):e488–95. Epub 2011/08/17. 10.1542/peds.2010-2825 ; PubMed Central PMCID: PMCPmc3164092. 21844053 PMC3164092

[33] Salafia C, Misra D, J. G, Platt C, Ring S. Characterization of Placental Growth as a Biomarker of Autism/ASD Risk International Meeting for Autism Research, Atlanta Georgia2014.

[34] Gardener H, Spiegelman D, Buka SL. Prenatal risk factors for autism: comprehensive meta-analysis. The British journal of psychiatry: the journal of mental science. 2009;195(1):7–14. 10.1192/bjp.bp.108.051672 19567888 PMC3712619

[35] Burstyn I, Sithole F, Zwaigenbaum L. Autism spectrum disorders, maternal characteristics and obstetric complications among singletons born in Alberta, Canada. Chronic diseases in Canada. 2010;30(4):125–34. Epub 2010/10/16. . 20946713

[36] Xu G, Jing J, Bowers K, Liu B, Bao W. Maternal diabetes and the risk of autism spectrum disorders in the offspring: a systematic review and meta-analysis. J Autism Dev Disord. 2014;44(4):766–75. Epub 2013/09/24. 10.1007/s10803-013-1928-2 ; PubMed Central PMCID: PMCPmc4181720. 24057131 PMC4181720

[37] Krakowiak P, Walker CK, Bremer AA, Baker AS, Ozonoff S, Hansen RL, et al Maternal metabolic conditions and risk for autism and other neurodevelopmental disorders. Pediatrics. 2012;129(5):e1121–8. Epub 2012/04/12. 10.1542/peds.2011-2583 ; PubMed Central PMCID: PMCPmc3340592. 22492772 PMC3340592

[38] Sankar KD, Bhanu PS, Ramalingam K, Kiran S, Ramakrishna BA. Histomorphological and morphometrical changes of placental terminal villi of normotensive and preeclamptic mothers. Anatomy & cell biology. 2013;46(4):285–90. Epub 2014/01/05. 10.5115/acb.2013.46.4.285 ; PubMed Central PMCID: PMCPMC3875846. 24386601 PMC3875846

[39] Mayhew TM, Ohadike C, Baker PN, Crocker IP, Mitchell C, Ong SS. Stereological Investigation of Placental Morphology in Pregnancies Complicated by Pre-eclampsia with and without Intrauterine Growth Restriction. Placenta. 2003;24(2):219–26. 10.1053/plac.2002.0900. 12566249

